# *COL17A1* editing *via* homology-directed repair in junctional epidermolysis bullosa

**DOI:** 10.3389/fmed.2022.976604

**Published:** 2022-08-25

**Authors:** Igor Petković, Johannes Bischof, Thomas Kocher, Oliver Patrick March, Bernadette Liemberger, Stefan Hainzl, Dirk Strunk, Anna Maria Raninger, Heide-Marie Binder, Julia Reichelt, Christina Guttmann-Gruber, Verena Wally, Josefina Piñón Hofbauer, Johann Wolfgang Bauer, Ulrich Koller

**Affiliations:** ^1^EB House Austria, Research Program for Molecular Therapy of Genodermatoses, Department of Dermatology and Allergology, University Hospital of the Paracelsus Medical University Salzburg, Salzburg, Austria; ^2^Cell Therapy Institute, SCI-TReCS, Paracelsus Medical University, Salzburg, Austria; ^3^Department of Dermatology and Allergology, University Hospital of the Paracelsus Medical University Salzburg, Salzburg, Austria

**Keywords:** gene editing, CRISPR/Cas9, homology-directed repair (HDR), junctional epidermolysis bullosa (JEB), *COL17A1*, gene therapy

## Abstract

**Background:**

Epidermolysis bullosa (EB), a severe genetic disorder characterized by blister formation in skin, is caused by mutations in genes encoding dermal-epidermal junction proteins that function to hold the skin layers together. CRISPR/Cas9-induced homology-directed repair (HDR) represents a promising tool for editing causal mutations in *COL17A1* in the treatment of junctional epidermolysis bullosa (JEB).

**Methods:**

In this study, we treated primary type XVII collagen (C17)-deficient JEB keratinocytes with either Cas9 nuclease or nickase (Cas9n) ribonucleoproteins (RNP) and a single-stranded oligonucleotide (ssODN) HDR template in order to correct a causal pathogenic frameshift mutation within the *COL17A1* gene.

**Results:**

As analyzed by next-generation sequencing of RNP-nucleofected keratinocytes, we observed an HDR efficiency of ∼38% when cells were treated with the high-fidelity Cas9 nuclease, a mutation-specific sgRNA, and an ssODN template. The combined induction of end-joining repair and HDR-mediated pathways resulted in a C17 restoration efficiency of up to 60% as assessed by flow cytometry. Furthermore, corrected JEB keratinocytes showed a significantly increased adhesive strength to laminin-332 and an accurate deposition of C17 along the basement membrane zone (BMZ) upon differentiation into skin equivalents.

**Conclusion:**

Here we present a gene editing approach capable of reducing end joining-generated repair products while increasing the level of seamless HDR-mediated gene repair outcomes, thereby providing a promising CRISPR/Cas9-based gene editing approach for JEB.

## Introduction

Genodermatoses, a heterogeneous group of inherited disorders mainly caused by mutations in genes encoding important structural proteins within the skin, have increasingly become a target for gene and cell therapies. Among these, gene therapy development for epidermolysis bullosa (EB) has been progressing rapidly, with a number of therapeutic applications being evaluated in clinical trials. EB is a rare genetic disease characterized by mucocutaneous fragility and blister formation following minor mechanical stress/trauma. Worldwide, about 500,000 people are affected by this phenotypically heterogeneous disorder, which is classified into four major subtypes according to the affected protein and level of disruption within the skin. Epidermolysis bullosa simplex (EBS), associated with intraepidermal blistering, is mainly caused by mutations within genes encoding keratin 5, 14, and plectin. Tissue separation below the lamina densa of the basement membrane zone (BMZ) is characteristic of the severe dystrophic form of EB (DEB), caused by mutations within the *COL7A1* gene, encoding type VII collagen (C7). The junctional form, JEB, is caused by mutations in genes encoding integrin-α6β4 (*ITGA6*, *ITGB4*), the integrin α3 subunit (*ITGA3*), laminin-332 (*LAMA3*, *LAMB3*, *LAMC2*), and type XVII collagen (C17, *COL17A1*), while Kindler syndrome is caused by mutations within the *KIND1* gene (1).

Gene therapy development for EB has thus far been dominated by strategies aiming to restore *COL7A1* and *LAMB3* function. The most clinically advanced and successful strategies are based on *in vivo* (*COL7A1*) (2) and *ex vivo* (*LAMB3*) (3–5) gene replacement using viral vectors. In contrast, development of gene therapies for *COL17A1*-associated JEB has been slow. Recent studies have revealed several interesting aspects of *COL17A1* biology in the context of skin homeostasis that make it an attractive target for *in vivo* gene therapy. In mice, a basal epidermal cell state, characterized by high expression of *Col17a1* and features of high quiescence and stemness, was recently identified (6). These cells could outcompete and eliminate their *Col17a1*-low and -negative counterparts. Subsequent application of scRNAseq, painted an emerging picture of the overall trajectory of *Col17a1^hi^* basal cells through several differentiation states in homeostasis, with enhanced cell fate plasticity and differentiation fluidity during wound healing (7). In the context of designing a gene therapeutic strategy, these studies suggest that: (i) 100% level of correction may not be necessary if the functionally corrected cells can eventually outcompete the C17-deficient population, and (ii) maintaining endogenous regulation of C17 as achieved by gene correction, as opposed to introduction of a constitutively expressed transgene, may be preferred.

In light of the above observations, we have focused on CRISPR/Cas9 gene editing strategies for the restoration of functional C17 protein in *COL17A1*-deficient cells. Gene editing *via* designer nucleases relies on the cleavage of DNA and subsequent activation of repair pathways in the cell. Double strand breaks generated are resolved either *via* classical or error-prone alternative end-joining (EJ) pathways (8), or by the more elegant homology-directed repair (HDR) pathway, which requires an externally provided homologous donor sequence (9–12). Remarkably, EJ-mediated gene disruption (13, 14) and gene reframing (15, 16) strategies currently demonstrate translational potential in terms of efficiency.

Recently, we developed the first gene editing strategy for functional correction of a *COL17A1* frameshift mutation in JEB that was based on induction of indel formation and reframing of the gene. The frameshift mutation, a 2-bp deletion (c.3899_3900delCT: formerly 4003delTC) in exon 52, was originally described as a common causal mutation in JEB (17). The strategy relied on deploying a pair of RNA-guided Cas9 nickases (D10A nickases) to induce two single-stranded nicks in close proximity to each other around the pathogenic mutation, generating a staggered DSB at the target site. Single DNA nicks are more likely to be repaired in a traceless manner, thereby greatly reducing potential adverse effects associated with off-target wild-type Cas9 nuclease activity (16). This proximal paired nicking (PPN) strategy resulted in expression of reframed C17 harboring short amino acid deletions, but that nevertheless demonstrated partial function, in ∼46% of treated JEB keratinocytes. Given the complexity of C17 biology, a perfect wild-type sequence restoration of the protein, which can be achieved *via* HDR, is still preferable.

Building on this previous work, we demonstrate improved repair outcomes with the addition of single strand oligonucleotide (ssODN) donor templates to direct HDR repair. While the addition of ssODN only marginally increased the outcomes from our previous PPN approach, treatment of primary JEB keratinocytes with high-fidelity (HiFi) Cas9 ribonucleoproteins (RNP) and ssODNs achieved an HDR efficiency of up to ∼38% on the genomic level, representing a significant improvement compared to previously reported efficiencies for the correction of EB-associated mutations.

## Materials and methods

### Cell culture and CRISPR system delivery

JEB keratinocytes were obtained from a skin biopsy of a patient bearing a homozygous frameshift mutation (c.3899_3900delCT) within exon 52 of *COL17A1* upon informed consent of the patient. Wild-type keratinocytes, which served as a positive control, were isolated from healthy donors upon informed consent. Healthy and patient primary keratinocytes were cultured in CnT-Prime Epithelial Proliferation Medium (CELLnTEC, Bern, Switzerland) at 37°C and 5% CO_2_ in a humidified incubator. One day before electroporation, cells were moved to 32°C and antibiotic-free medium. 3 × 10^5^ cells were electroporated in suspension with RNPs (3 μg HiFiCas9/Cas9n + 750 ng sgRNA) and ssODN (250 ng) using the Neon transfection system (Thermo Fisher Scientific, Waltham, MA, United States) at 1,400 V, 20 ms, 2 pulses. Transfected cells were maintained at 32°C for an additional 24 h before moving to 37^°^C.

### Semi-quantitative RT-PCR

RNA was isolated using the innuPREP RNA Mini Kit 2.0 (Analytik Jena, Jena, Germany), and cDNA was synthetized using the LunaScript RT SuperMix Kit (New England Biolabs, Ipswich, MO, United States) according to the manufacturer’s protocol. sqRT-PCR was performed with the Luna Universal qPCR Master Mix (New England Biolabs, Ipswich, MO, United States) using the CFX96 Touch Real-Time PCR Detection System (Bio-Rad, Hercules, CA, United States). *GAPDH* expression was used as housekeeping gene. Primers used for PCRs are listed in Supplementary Table 1.

### Flow cytometry

After washing twice with PBS and blocking with 10% sheep serum (Sigma-Aldrich, St. Louis, MO, United States) for 10 min, cell suspensions were incubated for 30 min at 4°C with polyclonal C17 antibody (Abcam, #184996, Cambridge, United Kingdom) as a primary antibody, diluted 1:1,000 in PBS. Following washing steps with PBS, cells were incubated with the secondary antibody (goat anti-rabbit FITC) (BD Biosciences, Franklin Lakes, NJ, United States) diluted 1:25 in PBS for another 30 min at 4°C in the dark. Cells were analyzed using the FACSAria III cell sorter (BD Biosciences, Franklin Lakes, NJ, United States). Data analysis was performed using the Kaluza software (Beckman Coulter, Brea, CA, United States).

### Protein isolation and western blot analysis

∼1 × 10^6^ cells were incubated with radioimmunoprecipitation assay (RIPA) buffer (Santa Cruz Biotechnology, Heidelberg, Germany) on ice for 30 min, and lysates were cleared by centrifugation at 20,000 × *g* for 30 min at 4^°^C. Cell culture supernatants were collected and filtered through 0.22-μm filters (TPP, Trasadingen, Switzerland). Proteins were precipitated overnight using ammonium sulfate, denatured at 95^°^C for 5 min in 4x loading buffer (0.25 M Tris, 8% SDS, 30% glycerol, 0.02% bromophenol blue pH 6.8), and resolved by electrophoresis in 8% Bis-Tris Plus gels. Following transfer to a nitrocellulose membrane, the membrane was blocked with blocking reagent (Roche Diagnostics GmbH, Mannheim, Germany) diluted 1:10 in Tris-buffered saline with 0.2% Tween (TBS-T) for 1 h at room temperature. The membrane was incubated overnight at 4°C with a C-terminal anti-C17 antibody (Abcam, #184996, Cambridge, United Kingdom) at a dilution of 1:1,000. β-tubulin (used as a loading control) was detected using a polyclonal antibody (Abcam, #6064, Cambridge, United Kingdom) diluted 1:2000 in TBS-T and blocking buffer. After 1 h incubation with a goat anti-rabbit HRP-labeled secondary antibody (Dako, Santa Clara, CA, United States) diluted 1:300 in TBS-T, visualization was performed with the Immobilon Western Chemiluminescent HRP Substrate (Merck, Darmstadt, Germany) and the ChemiDoc XRS Imager (BioRad, Hercules, CA, United States).

### Immunofluorescence staining of monolayers

1 × 10^5^ cells were seeded into 8-well chamber slides (ibidi, Gräfelfing, Germany). Cells were fixed with 4% formaldehyde (SAV Liquid Production, Flintsbach am Inn, Germany) for 10 min at room temperature. After washing twice with PBS, fixed cells were incubated for 2 h with a primary C-terminal anti-C17 antibody (Abcam, #184996, Cambridge, United Kingdom) diluted 1:1,000 in 1x blocking reagent (Roche Diagnostics GmbH, Mannheim, Germany) in PBS. For intracellular C17 detection (Supplementary Figure 1) 0.3% Triton X-100 was added. Following two washing steps with PBS, cells were co-stained with Alexa Fluor 488 goat anti-rabbit IgG (H + L) secondary antibody (Thermo Fisher Scientific, Waltham, MA, United States) diluted 1:400 and DAPI (4’,6-diamidino-2-phenylindole) (Thermo Fisher Scientific, Waltham, MA, United States) diluted 1:1000 in PBS, for 1 h at room temperature. Samples were analyzed using the confocal laser scanning microscope Axio Observer Z1 attached to LSM700 (Zeiss, Oberkochen, Germany).

### Generation of skin equivalents and immunofluorescence staining

5 × 10^4^ fibroblasts were immersed in a scaffold consisting of DMEM with 20% FCS, fibrinogen (F4883; Sigma-Aldrich, St. Louis, MO, United States) dissolved in 0.9% NaCl to a final concentration of 25 mg/ml, thrombin (T8885; Sigma–Aldrich, St. Louis, MO, United States) dissolved in 25 mM CaCl_2_, and aprotinin (A6279; Sigma–Aldrich, St. Louis, MO, United States). The scaffold was directly prepared in Falcon permeable support inserts with a 0.4 μm transparent PET membrane (Corning, New York, United States) and placed in BioCoat Deep-Well Plates (6-well, Corning, New York, United States) for 1 h at 37°C and 5% CO_2_. 2 × 10^5^ keratinocytes per well were seeded onto the matrix and grown to confluence in DMEM:Ham’s F-12 Green’s keratinocyte medium. Skin equivalents were then raised to the air-liquid interface and cultured for 21 days to allow for epidermal stratification. Cryosections of 8 μm were fixed with acetone:methanol (1:1) at −20°C and incubated for 2 h with a primary C-terminal anti-C17 antibody (Abcam, #184996, Cambridge, United Kingdom) diluted 1:1,000 in 1x blocking reagent (Roche Diagnostics GmbH, Mannheim, Germany) in Tris-buffered saline with 0.2% Tween (TBS-T). Visualization of the staining was performed using Alexa Fluor 488 goat anti-rabbit IgG (H + L) secondary antibody (Thermo Fisher Scientific, Waltham, MA, United States) diluted 1:400 and DAPI (4′,6-Diamidin-2-phenylindol) (Thermo Fisher Scientific, Waltham, MA, United States) diluted 1:1,000 in PBS, for 1 h at room temperature. Skin sections were analyzed using the confocal laser scanning microscope Axio Observer Z1 attached to LSM700 (Carl Zeiss).

### Next-generation sequencing preparation and analysis

Kapa HiFi Hot Start Ready Mix (Roche, Basel, Switzerland) was used for the amplification of genomic on- and off-target regions. Primers were designed specifically for each region and include adapter sequences for index-PCRs further downstream. These PCRs were then purified with Kapa Pure Beads (Roche, Basel, Switzerland) following the manufacturer’s protocol. PCR fragments below 200 bp were excluded *via* the right Amplicon-Bead ratios. The purified PCRs were then further amplified with Nextera XT Index Kit v2 Set A (Illumina, San Diego, CA, United States) and Kapa Hifi Hot Start Ready Mix. Similar to before, the second PCR was purified with Kapa Pure Beads (Roche, Basel, Switzerland), using the same Amplicon-Bead ratio as before. After quantification with the Qubit 2 Fluorometer and the Qubit dsDNA HS Assay Kit (Thermo Fisher Scientific, Waltham, MA, United States), samples were sent to the Vienna Biocenter Next Generation Sequencing Facility (VBCF, Vienna, Austria) for sequencing with the Illumina MiSeq Nano PE25 device. We generally aimed for 10,000–20,000 reads/sample.

After sequencing of amplicons, the generated files were quality controlled and converted to the appropriate format for analysis. This was carried out using the Galaxy platform (18).^1^ Downstream sequence analysis was performed with the CRISPR RGEN Cas-Analyzer tool (19).^2^

### Adhesion assay

96-well plates were coated with laminin-332 (10 μg/ml) (BioLamina AB, Sundbyberg, Sweden) overnight at 4°C. The wells were washed with PBS and blocked with 1% BSA for 1 h at 37°C. After another washing step, 3 × 10^4^ cells/well were then seeded on the pre-coated (10 μg/ml) 96-well plates and left to attach for 1 h at 37°C and 5% CO_2_ in a humidified incubator. After washing with PBS, cells were labeled with 4 μM CellTrace CalceinTM Green (Thermo Fisher Scientific, Waltham, MA, United States) in PBS for 90 min. Cells were gently washed two times with PBS and fluorescence intensities were measured using a Tecan microplate reader (Tecan Trading AG, Männedorf, Switzerland) at 485 nm excitation and 535 nm emission. The assay plates were then placed on an orbital shaker for 1 h at 1,000 rpm, followed by gently washing with PBS to remove detached cells. Subsequently, the fluorescence intensities of the adherent cell fractions were measured and expressed as percentages of fluorescence intensities measured before shaking.

### Colony formation assay

After electroporation of primary keratinocytes with the respective RNPs, cells were cultivated in 1 ml medium without antibiotics. After 20 min cells were seeded into 60-mm cell culture dishes pre-coated with rat type I collagen (Sigma-Aldrich, St. Louis, MO, United States). After 24 h at 37°C and 5% CO_2_, 3–5 × 10^5^ mitomycin C-treated 3T3-J2 feeder cells were added. Feeder cells were prepared by treating 3T3-J2 mouse fibroblasts with 4 μg/ml mitomycin C for 2 h, followed by washing with PBS. Keratinocytes were maintained on feeder cells for 14 days until colonies were visible. Before fixing and staining the cells supernatants were harvested for Western blot analysis of restored and shedded C17 protein. Feeder cells were trypsinized and discarded before staining the colonies. Colonies were fixed in 4% formaldehyde and stained with Rhodamine B (Sigma–Aldrich, St. Louis, MO, United States). Colony growth areas were calculated *via* ImageJ (20).^3^

### Statistical analysis

Unpaired student’s *t*-tests as well as one-way ANOVA (with the appropriate multiple comparisons test) were performed using GraphPad Prism (GraphPad Software, La Jolla, CA, United States). *P*-values (Significances): > 0.05 (not significant), ≤ 0.05 (^∗^), ≤ 0.01 (^∗∗^), ≤ 0.001 (^∗∗∗^).

## Results

### Homology-directed repair strategy for *COL17A1* editing

**FIGURE 1:**
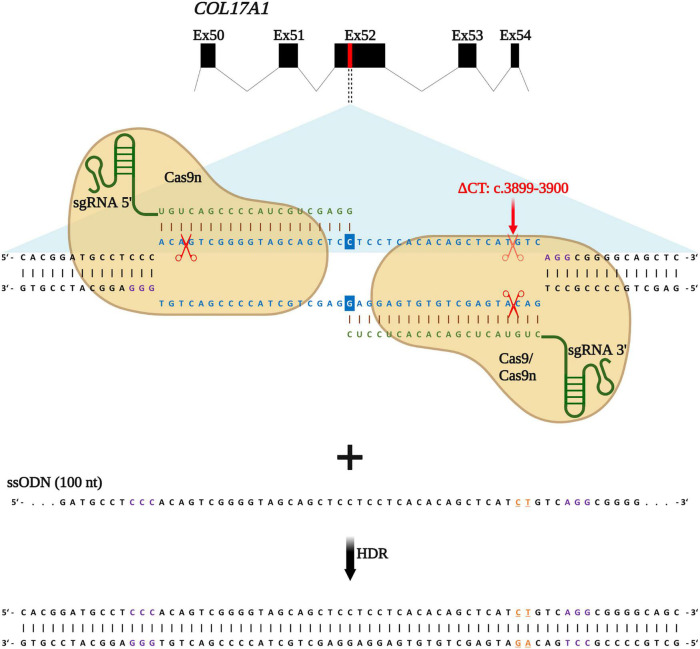
HDR-based strategy for *COL17A1* repair. sgRNA 3′ is specific for the c.3899_3900delCT mutation within exon 52 while sgRNA 5′ guides Cas9n (for paired nicking) to cut proximal to the mutation. sgRNAs (green) guide Cas9 or Cas9n (for paired nicking) to cut upstream of the PAM sequence (violet). Single-stranded oligonucleotides (ssODN) serve as repair templates carrying the wild-type *COL17A1* sequence including the nucleotides C and T (orange letters) that are absent in JEB patient cells. Created with BioRender.com.

We recently reported an efficient strategy for gene editing of a homozygous frameshift mutation in *COL17A1* exon 52 (c.3899_3900delCT) based on gene reframing (16). While this strategy resulted in heterogenous indel patterns on the genomic level, only a few predominant transcripts, which encoded in-frame C17 proteins bearing short amino acid (aa) deletions, emerged. This resulted in a largely homogenous repair outcome and protein restoration with only minimal aa divergences to the wild-type C17 protein. However, the ultimate aim of gene therapy is the traceless repair of disease-causing mutations, which can be achieved through HDR. In a previous work, we demonstrated that the use of ssODN with homology arms of 50 nucleotides in length, complementary to the target locus, was more efficient in terms of precision and safety compared to dsODNs (12). We therefore designed a similar HDR-based gene editing strategy to accurately correct the above-mentioned *COL17A1* mutation in JEB keratinocytes using our previously validated sgRNAs (16). sgRNA 3′ binds to and induces DNA cleavage directly at the mutation site, whereas sgRNA 5′ binds and cleaves in close proximity (within 33 nts) upstream of the mutation (Figure 1).

Following induction of DNA breaks, various repair pathways are co-opted based on several factors, such as cell cycle state and availability of repair templates. Building upon our previous repair strategy, we investigated whether the addition of the ssODN could significantly shift repair from end-joining (EJ) to HDR. RNPs, consisting of Cas9 nickases complexed with both sgRNA 5′ and sgRNA 3′, were delivered into primary JEB keratinocytes *via* electroporation with or without the ssODN template. We also delivered Cas9 nuclease/sgRNA 3′ RNP ± ssODN for comparison. We employed the HiFiCas9 nuclease (Integrated DNA Technologies: IDT), recently shown to exhibit improved specificity over wild-type Cas9, demonstrating a further improvement on our previous work.

### *COL17A1* gene editing outcomes in primary junctional epidermolysis bullosa keratinocytes

**FIGURE 2:**
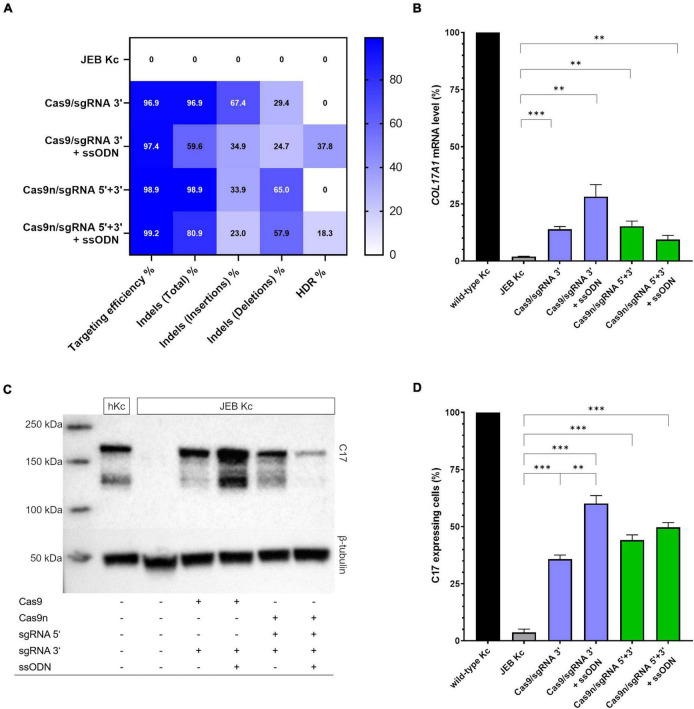
*COL17A1* editing outcome in RNP and ssODN-treated primary JEB keratinocytes. **(A)** NGS analysis of the *COL17A1* on-target locus presented as a heat map showing indel and HDR pattern of the performed editing strategies. *n* = 2. Mean value **(B)** sqRT-PCR analysis of *COL17A1* mRNA expression in JEB keratinocytes upon RNP/ssODN treatment. Primary wild-type and untreated JEB cells served as controls. *n* = 3; Mean ± SEM; Student’s two-tailed *t*-test. **(C)** Western blot analysis of cell lysates revealed the presence of restored C17 (180 kDa). β-tubulin (50 kDa) served as loading control. **(D)** Flow cytometric analysis of C17-stained JEB keratinocytes revealed a high level of C17 restoration. *n* = 3; Mean ± SEM; Student’s two-tailed *t*-test. *P*-values (Significances): > 0.05 (not significant), * ≤ 0.05, ** ≤ 0.01, and * ≤ 0.001.

We employed next-generation sequencing (NGS)-based analyses to evaluate the repair outcomes achieved with the various editing strategies. Our analyses revealed that the overall targeting efficiency, calculated as the sum total of edits, was consistently over 96% at the predicted position with every combination tested (Figure 2A). More importantly, we examined if repair outcomes were induced *via* EJ or HDR. In exclusively RNP-treated JEB keratinocytes (i.e., without co-delivery of ssODN), ∼97% (Cas9/sgRNA 3′) and ∼99% (Cas9n/sgRNA 5′ + 3′) of NGS-analyzed *COL17A1* alleles harbored indels, which are predominantly induced *via* the EJ repair pathway. As expected from our previous work (16), deletions were more frequently induced in JEB keratinocytes treated with Cas9n/sgRNA 5′ + 3′, as compared to those treated with the Cas9/sgRNA 3′ combination. Co-delivery of ssODNs with the RNPs into JEB keratinocytes resulted in 37.8 and 18.3% of HDR when employing Cas9/sgRNA 3′ and Cas9n/sgRNA 5′ + 3′, respectively (Figure 2A). The ratio of alleles harboring indels to alleles corrected in a traceless manner was ∼1.5:1 in Cas9/sgRNA 3′/ssODN-treated cells and ∼4.4:1 in Cas9n/sgRNA 5′ + 3′/ssODN-treated cells. Thus, regardless of the editing molecules used, the co-delivery of ssODN template could direct a shift from EJ to HDR induction. Especially when using Cas9 RNPs, the shift toward HDR-mediated traceless correction was more prominent.

Besides the analysis of on-target editing efficiency, we also assessed the presence of gene edits at selected off-target (OT) sites recently identified for both sgRNAs (16). In our previous work, 15 OT sites each were predicted *via* various *in silico* tools for both 5′- and 3′- sgRNAs. Of the total 30 OT sites, we detected edits at significant frequencies of 5.32, 32.00, and 4.77% at 5′OT1, 5′OT3, and 3′OT11, respectively, in cells treated with RNP complexes of wild-type Cas9 and the corresponding sgRNA (16). Therefore, we specifically interrogated these three high-risk OT sites for the presence of any off-target edits, including integration of the ssODN. Our results showed no evidence of off-target ssODN integration or any other off-target event detectable *via* NGS (Supplementary Figure 2). Thus, we could confirm our previous results demonstrating the safety of employing paired Cas9n RNPs in a proximal nicking approach, and additionally demonstrate here the enhanced specificity of HiFi Cas9 nuclease over the wild-type variant.

We further verified *COL17A1* restoration *via* semi-quantitative RT-PCR (sqRT-PCR), revealing a significant increase in *COL17A1* mRNA levels in JEB keratinocytes in all four treatment conditions (Figure 2B). By this analysis, treatment with Cas9/sgRNA 3′ + ssODN gave the best restoration rates, achieving *COL17A1* mRNA levels of ∼28% that of healthy control keratinocytes. Western blot analysis revealed the restoration of full-length C17 in whole cell lysates of treated JEB keratinocytes (Figure 2C and Supplementary Figure 3). Remarkably, the shed C17 ectodomain was absent in untreated JEB cells, but detectable in the supernatants of CRISPR/Cas9-treated JEB keratinocytes (Supplementary Figure 4).

**FIGURE 3:**
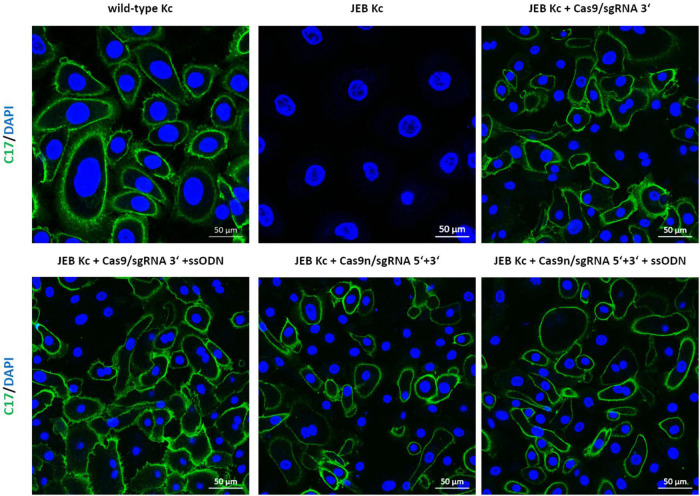
Immunofluorescence staining of RNP/ssODN-treated primary JEB keratinocytes. Immunofluorescence staining of C17 (green) revealed the accurate deposition of the restored protein in RNP and ± ssODN-treated primary JEB keratinocytes. Nuclei were stained with DAPI (blue).

Analysis of C17 surface expression by flow cytometry revealed ∼ 60 and 50% of C17-positive JEB cells upon co-delivery of the ssODN HDR template with Cas9/sgRNA 3′ and Cas9n/sgRNA 5′ + 3′, respectively (Figure 2D and Supplementary Figure 5). Furthermore, the accurate localization of reframed C17 protein variants within the cell membrane was observed *via* immunofluorescence analysis (Figure 3). Taken together, Cas9/sgRNA 3′ + ssODN treatment of JEB keratinocytes showed the most promising *COL17A1* repair profile at the DNA-, RNA-, protein-, and cellular levels. The addition of ssODN HDR template in addition to proximal paired nicking using Cas9n/sgRNA5′ + 3′ only marginally improved the overall repair outcome at cellular levels.

### Analysis of the adhesive strength of corrected junctional epidermolysis bullosa keratinocytes to laminin-332

**FIGURE 4:**
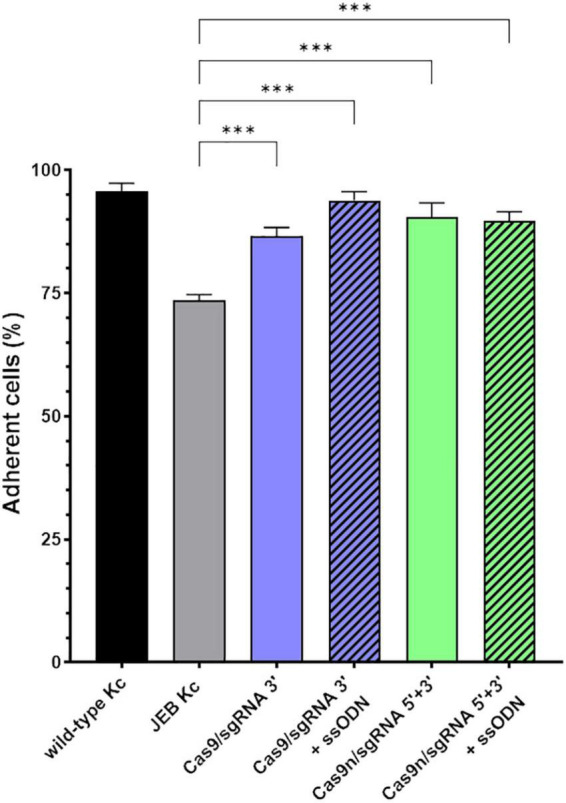
Adhesive properties of RNP-treated primary JEB keratinocytes. Adhesion analysis revealed higher adhesive strength of RNP and ± ssODN-treated primary JEB keratinocytes to laminin-332 compared to untreated JEB control. *n* = 3, Mean ± SEM; Student’s two-tailed *t*-test. *P*-values (Significances): > 0.05 (not significant), * ≤ 0.05, ** ≤ 0.01, and *** ≤ 0.001.

Since laminin-332 is a major binding partner of C17 within the BMZ of the skin (21), we analyzed the adhesive strength of restored C17 to laminin-332, as the targeted *COL17A1* mutation impairs the interaction of these structural proteins (16) (Figure 4). Fluorescently labeled cells bound to a laminin-332-coated surface were subjected to shear forces (shaking at 1,000 rpm for 1 h) in order to detach cells from the laminin-332 surface. In any treated JEB keratinocyte sample we observed a significantly higher percentage of cells that remained bound to the coated surface compared to untreated cells (Figure 4). This is apparent in JEB cells treated exclusively with RNPs, which leads to reframed but slightly truncated C17 variants as recently demonstrated (16), and in JEB cells in which ssODNs were co-delivered with the RNPs. In the latter, a fraction of the bulk-treated keratinocyte population expresses wild-type C17 protein, as HDR generates a traceless repair. Interestingly, this experiment showed no clear advantage of (low-level) HDR-mediated wild-type C17 restoration with respect to C17 function. However, future experiments using xenograft transplantation models will reveal the impact of EJ- and HDR-based *COL17A1* repair on long-term skin integrity, as well as longevity, including survival and proliferation advantages of the various gene-corrected clones.

### Impact of gene editing procedure on colony forming capacity

**FIGURE 5:**
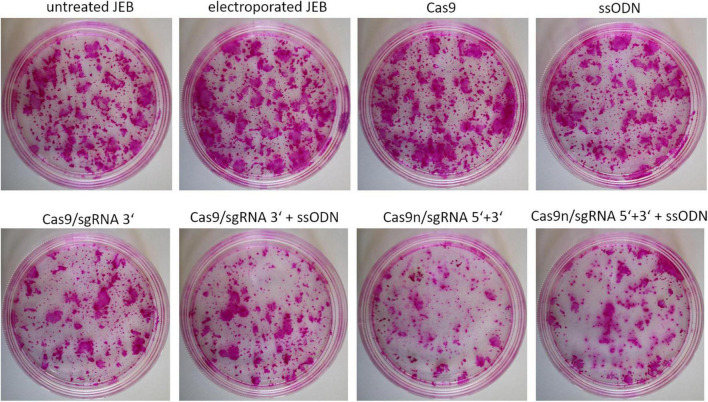
Colony-forming capacity of treated JEB keratinocytes. Rhodamine stainings of JEB clones electroporated with and without Cas9 nuclease (without sgRNAs) showed no impact on cell viability and colony forming capacity. However, using either Cas9 or Cas9n with sgRNAs had a significant impact on clonal capacity of the treated cells.

In view of the intended development of our gene editing strategies for future clinical application in JEB, we analyzed the colony-forming capacity of primary keratinocytes after treatment. Treated primary JEB keratinocytes were analyzed *via* immunofluorescence staining revealing a high C17 restoration efficiency for each gene editing strategy (Supplementary Figure 1). Colony formation assays demonstrated that the electroporation of cells with the HiFi Cas9 nuclease alone (without sgRNA) had no obvious negative effect on colony-forming capacity (Figure 5). However, a strong effect after RNP (w/wo ssODN) treatment was detected, suggesting a critical cellular stress level induced *via* genomic cleavage/editing. Especially following treatment with paired nicking, the area of clonal growth was reduced to 35.8% of that detectable in untreated JEB keratinocytes (Supplementary Table 2).

### C17 deposition in 3D skin equivalents

**FIGURE 6:**
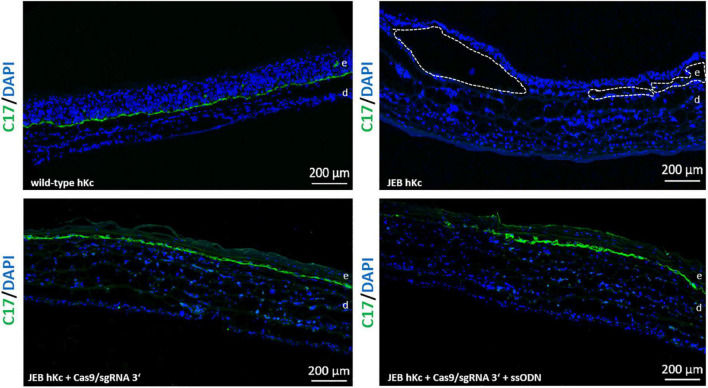
C17 immunofluorescence staining of 3D skin equivalents. 3D skin equivalents from wild-type and JEB keratinocytes served as positive and negative control, respectively. The dotted line in white in the negative control indicates blisters within the BMZ. Epidermis and dermis are marked with “e” and “d.” Images show 3 × 3 tile scans of the 10x objective. C17 (green), DAPI (blue).

To investigate correct C17 deposition at the BMZ, we generated 3D skin equivalents from wild-type, untreated and treated primary JEB keratinocytes. Immunofluorescence analysis of cryosections of skin equivalents expanded from Cas9/sgRNA 3′ + ssODN-treated cells revealed accurate C17 deposition at the BMZ. In contrast, C17 was completely absent in skin equivalents from untreated JEB keratinocytes (Figure 6).

## Discussion

So far, the traceless repair of disease-associated mutations in EB *via* HDR has been associated with low editing efficiencies and, critically, the induction of off-target edits. Together with the necessity for exogenous repair templates and the restriction of activity in dividing cells, these features had previously reduced the therapeutic potential of HDR-based approaches. The HDR-repair outcomes achieved with the combination of HiFiCas9 RNPs and ssODN repair templates in this study represent a significant improvement over past efforts in the traceless correction of EB mutations, both in terms of efficiency and safety. The introduction of Cas9 nucleases as RNPs for gene editing limits the potential for off-target effects, as ongoing protein degradation shortens the time window of activity of the nuclease. This is expected to translate into significantly improved safety profiles, as compared to strategies relying on long-term expression of the nuclease from plasmids. Even so, our previous application of wild-type Cas9 RNPs resulted in detectable off-target activity, which could be abrogated by the use of Cas9 nickases in a PPN approach, or by the use of HiFiCas9 nuclease, which displays superior specificity compared to the wild-type nuclease. In general, the targeting efficiency (i.e., DSB generation) was equivalent for both the PPN approach and HiFiCas9, and can be calculated from the frequency of indels generated (>96% for both). This also represents the efficiency of EJ-mediated repair. The addition of ssODNs successfully enabled the activation of HDR and a competitive shift from the use of the error-prone alternative EJ mechanism to the more elegant HDR pathway. At the *COL17A1* locus investigated here, the staggered DSB generated by paired nicking proved to be a less efficient inducer of HDR as compared to the blunt DSB generated by the HiFiCas9 nuclease, as reflected in the total HDR-repair frequencies observed (∼18% vs. ∼38%).

Statistically, only one-third of indels generated are expected to restore the C17 reading frame. We can infer the level of functional C17 restoration achieved from our flow cytometry measurements, as correct cell surface expression requires correct protein folding and transport to the cell membrane. According to these analyses, > 60% of cells treated with HiFiCas9/sgRNA 3′ + ssODN expressed C17 at their cell surface. This represents a significant increase by ∼25% over EJ-mediated repair alone, and is the highest correction efficiency at this locus achieved to date, accounting for the total functional correction derived from the combination of EJ- and HDR-repair. In contrast, the combination of paired nicking and ssODN did not significantly enhance total functional C17 levels (∼50%) as achieved *via* PPN alone in our recent study (16). Even so, successful HDR-repair means that a fraction of the total C17 protein measured should be fully functional wild-type protein, potentially contributing to overall improved functional outcomes and skin stability. Surprisingly, a similarly increased adhesive strength to laminin-332 compared to untreated JEB cells could be observed in our adhesion assays, regardless of which gene editing strategy was applied. This could reflect a limitation in the robustness of this *in vitro* assay. Theoretically, every change in the amino acid sequence of C17 can impact its function, which can be assessed in stability experiments performed in long-term skin models. Indeed, future studies will focus on evaluating divergences in stability imparted by wild-type vs. reframed C17 variants in suitable animal models. Nevertheless, any strategy that increases expression of the wild-type protein albeit at a low level is preferable, as the wild-type protein excludes any concerns regarding potential toxicity and lack of functionality of truncated C17 variants. While we observed the accurate and nearly continuous deposition of C17 along the epidermal/dermal junction within skin equivalents derived from corrected (∼60% C17 positive cells) primary JEB keratinocytes, an in-depth analysis of skin integrity is best performed in a xenograft mouse model. Such analyses will provide invaluable insight into the long-term and clonal outcomes of the gene repair, particularly, if C17 restoration imparts a competitive advantage and/or maintains stemness in corrected cells.

Besides the adhesion to laminin-332 we also analyzed the cell *via*bility and colony-forming potential of RNP/ssODN-treated JEB keratinocytes, revealing a significant impact of the respective gene editing procedures. This has to be considered for future clinical studies. We observed no integration of ssODNs (12), neither at cleaved on- nor off-target sites, legitimating the development of new technologies employing traceless gene editing. In recent years, base editors have been steadily improved in order to introduce four possible transition mutations, thereby bypassing the requirement for DSB induction or donor DNA templates (22, 23). In another form of EB, namely, recessive dystrophic EB (RDEB), the repair of *COL7A1* mutations in primary fibroblasts using this technology was shown, with efficiencies of up to 45% on mRNA levels achieved (24). Prime editing constitutes another seminal gene editing method successfully applied by Hong et al. to the correction of representative *COL7A1* mutations with efficiencies ranging from 5.2 to 10.5% on the genomic level and up to 46% on the protein level (25).

To date, the most efficient and potentially safest gene therapy relies on the *ex vivo* correction of gene function, successfully shown for various genetic diseases including the skin. For JEB, *LAMB3* cDNA replacement therapy has been conducted in three patients, leading to successful long-term clinical outcomes and increased quality of life (3–5). As a cDNA replacement therapy is currently not available for JEB with *COL17A1* deficiency, we present here a promising approach for the *ex vivo* correction of junctional EB based on efficient CRISPR/Cas9-based induction of HDR in combination with EJ-based gene reframing. Although the exact influence of indels generated at the targeting site on therapeutic outcomes needs to be thoroughly analyzed in future studies and may prove to be negligible for achieving skin integrity, the restoration of fully functional wild-type protein is still preferable. Currently, this can be achieved *via* HDR, base editing, or prime editing. Notably, the efficient HDR strategy used here can be adapted for correcting any pathogenic mutation in *COL17A1* in the future, in contrast to EJ-mediated gene reframing, whose application is limited to the correction of frameshift mutations. Most importantly, a suitable technique has to combine high efficiencies with an assessable safety profile, as is the case with the current HDR approach presented here.

## Data availability statement

The datasets generated for this study can be found in the zenodo database: Illumina MiSeq PE250 Nano on-target data for HDR-treated primary keratinocytes as well as off-target data for sgRNA 5′ and 3′ (https://doi.org/10.5281/zenodo.6683613).

## Author contributions

UK, OM, JR, and JWB were involved in the conception and design of the study. IP, JB, TK, OM, BL, SH, AMR, H-MB, CG-G, VW, and JP were involved in data generation, analysis, and interpretation. JR, OM, DS, JWB, and UK provided funding for the study. UK, IP, and JP wrote the manuscript. UK supervised the whole study. All authors were involved in manuscript editing.

## Footnotes

1
https://usegalaxy.org

2
http://www.rgenome.net/cas-analyzer/#!

3
https://imagej.nih.gov/ij/docs/index.html
