# Supplementary material for: COL17A1 editing via homology-directed repair in junctional epidermolysis bullosa
Source: Front Med (Lausanne). 2022 Aug 25;9:976604. doi: 10.3389/fmed.2022.976604 (PMC9454317; doi:10.3389/fmed.2022.976604)
Supplement: Supplementary file 1 [file Data_Sheet_1.pdf]

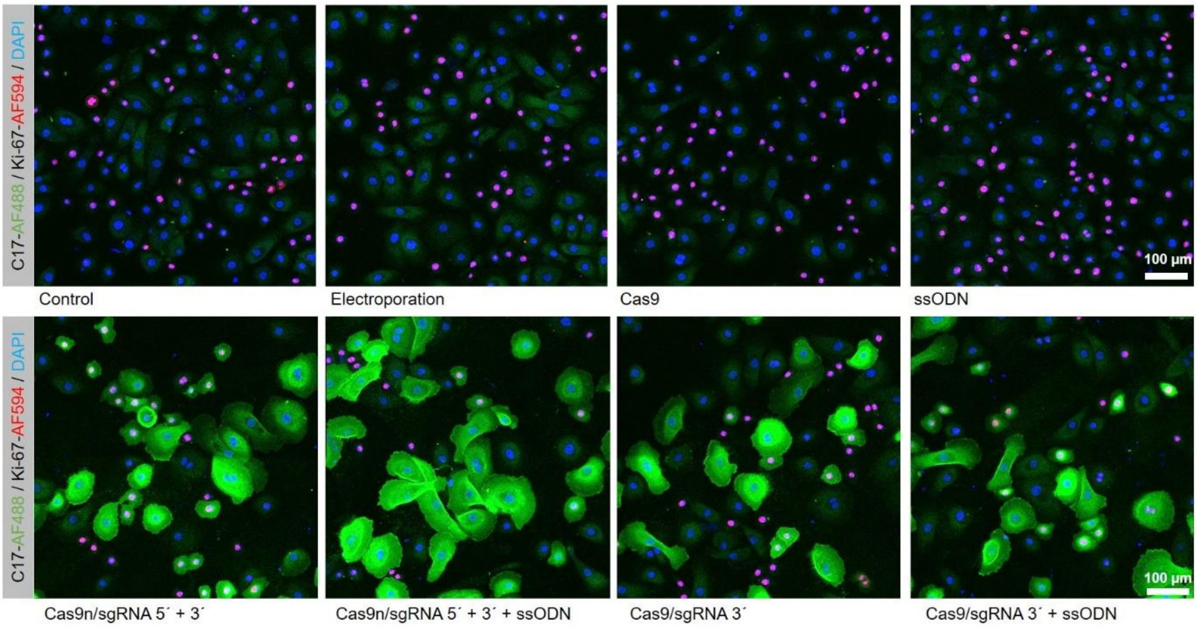

Figure S1: Immunofluorescence staining prior to colony formation assay. IF analysis prior to seeding of treated JEB keratinocytes onto feeder layer for colony formation analysis revealed a high C17 restoration efficiency for all gene editing strategies.

sgRNA	On/Off-target	Protospacer	PAM	Gene	Locus
sgRNA 3'	on-target	C T C C T C A C A C A G C T C A T G T C	A G G	COL17A1	chr10:-105,794,014
	OT11	T . . . . G . . 2 . 2 . . . . - -	G . .	B2M	ch15:+45,003,716
sgRNA 5'	on-target	G G A G C T G C T A C C C C G A C T G T	G G G	COL17A1	chr10:+105,793,977
	OT1	A . . A . . . T . . . . . . A	T . .	Intergenic region	chr1:+188,318,688
	OT3	T . . . . T	. . .	Intergenic region	chr17:+13,005,412

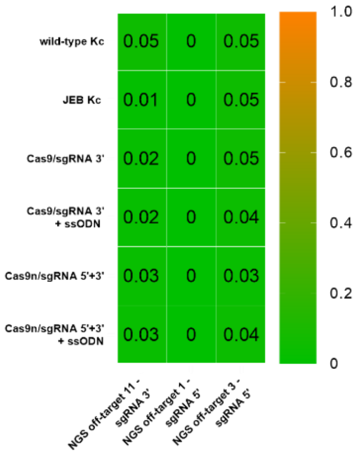

Figure S2: Analysis of off-target indel formation and ssODN integration. NGS-based analysis of previously identified off-target sites for sgRNA 3' (OT11) and sgRNA 5' (OT1 and OT3) revealed no signs of indel formation and ssODN integration.

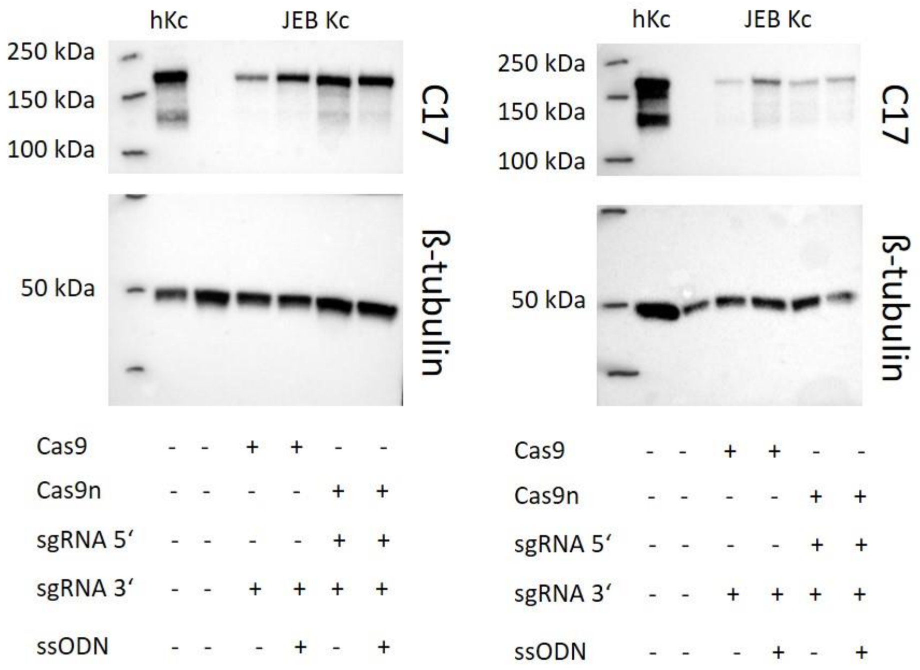

Figure S3: Western blot analysis of cell culture lysates
Western blot analysis of cell culture lysates of RNP (w/wo ssODN)-treated primary JEB keratinocytes revealed the presence of full-length C17 at a size of 180 kDa.

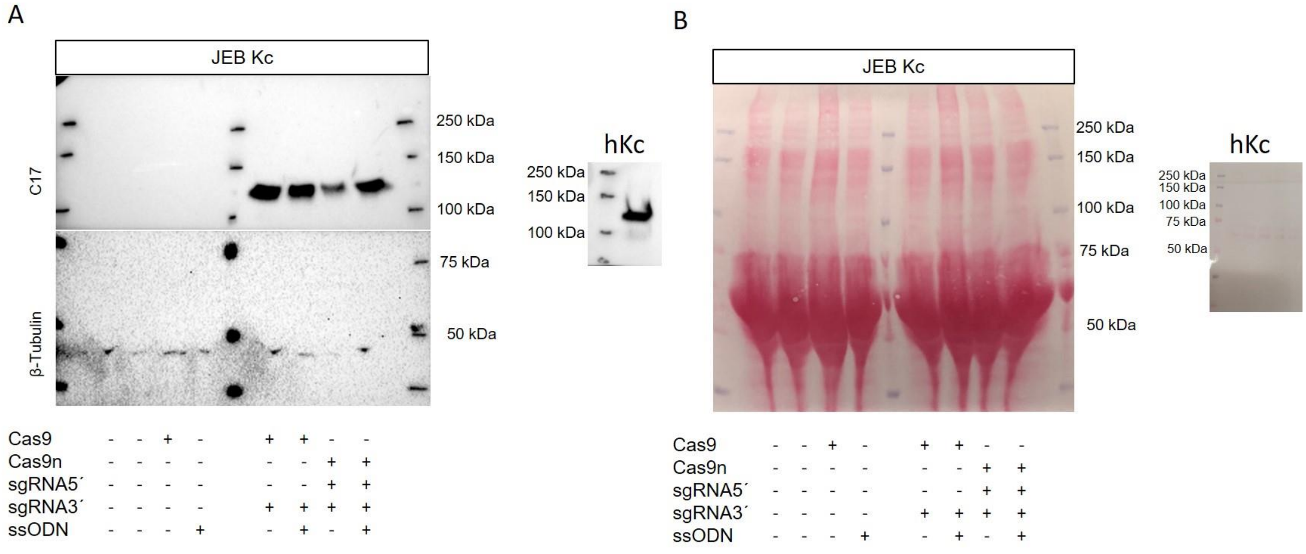

Figure S4: Western blot analysis of cell culture supernatants. (**A**) Western blot analysis of supernatants of RNP (w/wo ssODN)-treated primary JEB keratinocytes and wild-type keratinocytes (hKc) revealed the presence of the C17 ectodomain at a size of 120 kDa. (**B**) Ponceau red staining of cell culture supernatants.

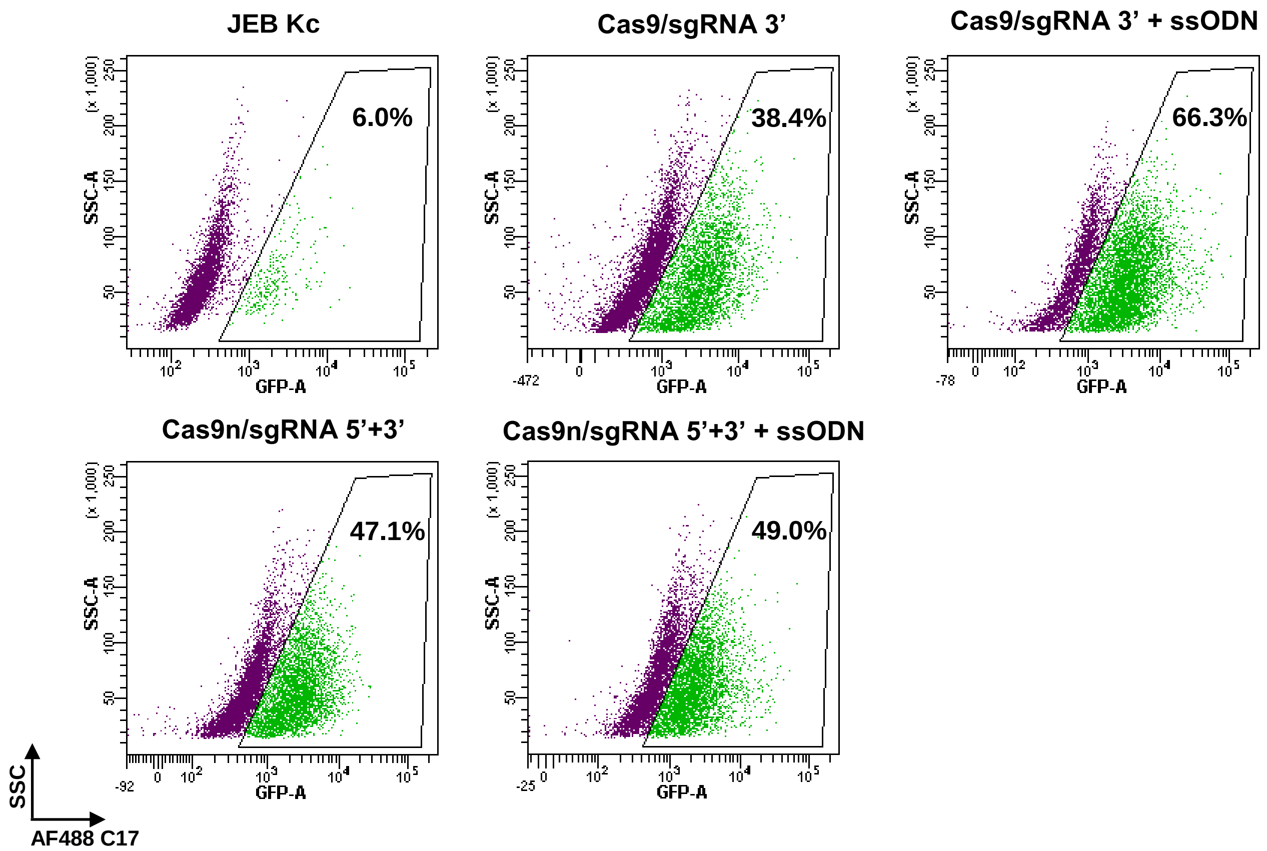

Figure S5: Gating strategy for flow cytometric analysis of C17-stained JEB keratinocytes.

	Forward primer (5’ – 3’)	Reverse primer (5’ – 3’)
PCR On-target	TAAGCCCGGTTTCCTTCCAC	TACGCTGCATGCTCTCTGAC
NGS On-target	TCGTCGGCAGCGTCAGATGTG TATAAGAGACAGCCCACTCAT GCAGCTTCTCACC	GTCTCGTGGGCTCGGAGATGTG TATAAGAGACAGCGATGTCAGT GCCATAGGGACC
NGS off-target 11 – sgRNA 3’	AAAACGGGAAAGTCCCTCTC	GAGAGACTCACGCTGTCACG
NGS off-target 1 – sgRNA 5’	CATTGCCACGAGTATTGCAT	CCCTGGGAAAAGATGTGACT
NGS off-target 3 – sgRNA 5’	GAGTTACTGCCCCTTTCCAT	CCACAAGGTTCTTGGCTTT
sqRT-PCR – *COL17A1*	GCAATGGCGGACTATTGGGA	AGTCACGTTGCTGTAGGCAG
sqRT-PCR – *GAPDH*	GCCAACGTGTCAGTGGTGGA	CACCACCCTGTTGCTGTAGCC
ssODN	C*A*C*GGATGCCTCCCACAGTCGGGGTAGCAGCTCCTCCTCACA CAGCTCATCTGTCAGGCGGGGCAGCTCCTACAGCTCTTCCATGAG CACAGGAGGAG*G*T*	

*Phosphorothioate backbone

Table S1: Primers used for PCR, NGS on-and off-target PCR and sqRT-PCR.

Treatment	relative clonal growth area [%]
Control	100,0
Electroporation	145,1
Cas9	126,6
ssODN	127,3
Cas9/sgRNA 3'	66,2
Cas9/sgRNA 3' + ssODN	52,6
Cas9n/sgRNA 5'+3'	35,8
Cas9n/sgRNA 5'+3' + ssODN	37,7

Table S2: Relative clonal growth area upon CRISPR/Cas9 treatment
